# Usefulness of microsurgical back‐table angioplasty for multiple hepatic arteries in living donor liver transplantation

**DOI:** 10.1002/ags3.12370

**Published:** 2020-07-06

**Authors:** Noboru Harada, Tomoharu Yoshizumi, Toshiharu Matsuura, Tomoaki Taguchi, Masaki Mori

**Affiliations:** ^1^ Department of Surgery and Science Graduate School of Medical Sciences Kyushu University Fukuoka Japan; ^2^ Department of Pediatric Surgery Graduate School of Medical Sciences Kyushu University Fukuoka Japan

**Keywords:** angioplasty, hepatic artery, liver transplantation, surgical anastomosis, vascular surgical procedures

## Abstract

The graft hepatic artery orifice is tiny in living donor liver transplantation, and therefore, it is more difficult to reconstruct the hepatic artery than in deceased donor liver transplantation. In situ, multi‐vessel hepatic artery reconstruction in living donor liver transplantation is time‐consuming, and reconstructions are often complicated if the hepatic graft has several stumps. We describe two living donor liver transplants using back‐table microsurgical angioplasty to combine two hepatic artery stumps to create a single orifice, and sequential single‐vessel hepatic artery reconstruction in the recipient. Briefly, we used double‐needle interrupted sutures for the two hepatic artery stumps with a biangular stay‐suture method in back‐table microsurgical angioplasty. Each suture was placed from the inner side of the arterial wall to the outer side, which allowed for safe and reliable suturing. After placing the interrupted sutures in the anterior wall, we turned over the vessels in the cold storage on the back table and placed interrupted sutures in the posterior wall. In the recipient, the single stump of the graft was anastomosed to the recipient's hepatic artery using an interrupted pattern and a surgical microscope. The postoperative courses of the donors and recipients were uneventful. Back‐table hepatic artery angioplasty is a feasible option to overcome the complexities of multi‐vessel arterial reconstruction in living donor liver transplantation. We recommend performing secure multi‐vessel hepatic arterial reconstruction adapted to the clinical scenario. Using simple appropriate anastomosis, back‐table microsurgical angiography may provide good results in living donor liver transplantation.

## Graphical abstract

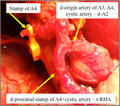

## 1 INTRODUCTION

In living donor liver transplantation (LDLT), many anastomotic variations of the hepatic artery require reconstruction. Although most left‐lobe grafts have a single artery, such as the left hepatic artery or replaced left hepatic artery, there are sometimes two or three arteries such as A2, A3, or A4. According to the Hiatt classification, in 1000 grafts,^1^ normal left hepatic arteries arose from the celiac axis to form the left hepatic artery branches in 876 grafts, while in some grafts, A2, A3, and A4 vessels originated individually from the proper hepatic artery,^2^ with an incidence of 3.4% in left liver grafts.^3^ In the right lobe, one or two arteries have been identified, such as anterior and posterior arteries. In a previous report of 96 LDLTs using a right‐lobe graft,^4^ most grafts had a single orifice (n = 185, 96%), but seven right livers (4%) had multiple arteries, namely, a replaced artery in five livers and accessory arteries in two livers. Three liver grafts had two separate orifices: both arterial stumps were reconstructed in one patient, and accessory arteries were ligated in two patients because of sufficient back flow. We recently reported that middle and left hepatic arterial reconstruction is safe in LDLT and may prevent biliary stricture caused by dual hepatic arterial reconstruction, when the graft has left and middle hepatic arterial stumps.^5^ To secure the anastomosis of the tiny dominant artery of the graft, back‐table angioplasty, which permits in situ reconstruction of only one hepatic artery anastomosis in the recipient operation, may be feasible because a single‐orifice hepatic artery in the graft is simpler and easier to anastomose with the recipient hepatic artery compared with multi‐vessel reconstruction. We present two liver transplantations using back‐table microsurgical angioplasty to combine the two hepatic artery stumps into one orifice, and reconstruction of the hepatic artery in the recipient surgery, in LDLT. In this report, we presented the details of two patients who underwent LDLT for whom each graft involved combining two hepatic artery stumps into one orifice using back‐table angioplasty.

## 2 METHODS

### 2.1 Case presentation 1

**Figure 1:**
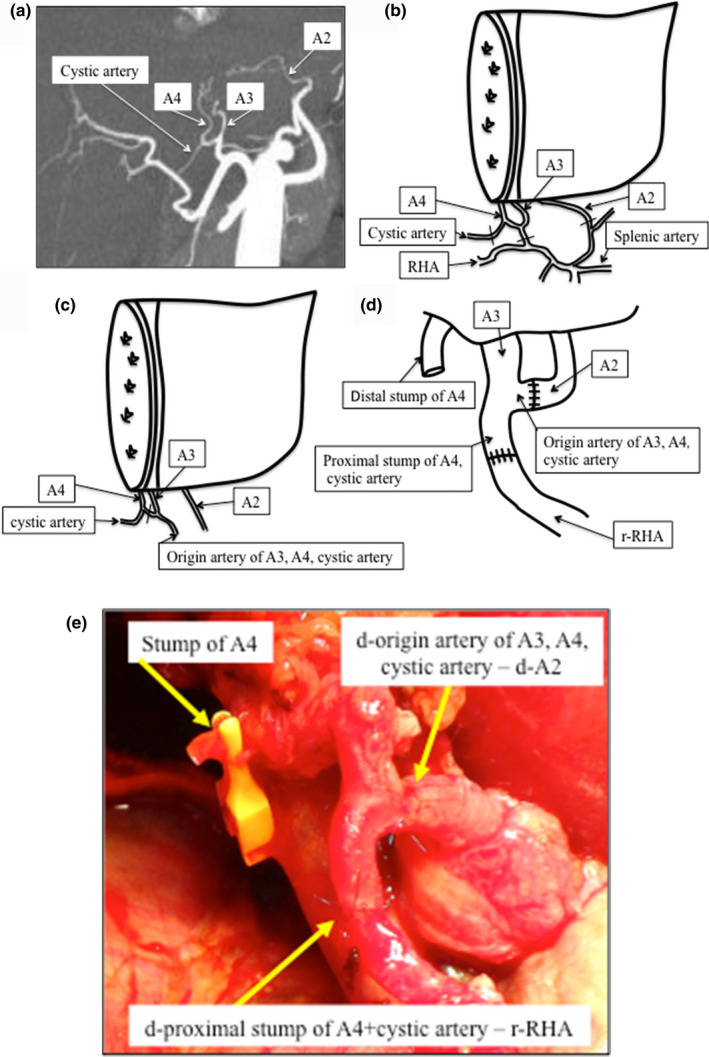
Imagings and schemas for Case 1. A, Three‐dimensional CT angiography of the hepatic artery (maximum intensity projection: MIP imaging). B, Schema for the hepatic artery of the hepatic graft. Each line is the division point for A2, A3, A4, and the cystic artery. A2, hepatic artery of segment 2; A3, hepatic artery of segment 3; A4, hepatic artery of segment 4; RHA, right hepatic artery. C, Schema for the dividing point (line) of the hepatic artery between A3 and A4 with cystic artery on the backtable after hepatectomy. D, Schema for the graft after angioplasty between the origin artery of A3, A4, cystic artery and A2 stump, and between proximal stump of A4, cystic artery and r‐RHA. The multiple lines across the vessels indicate the anastomosis point of the reconstructed hepatic artery. r‐RHA, recipient's right hepatic artery. E, Intraoperative photograph of the reconstructed hepatic artery (donor‐proximal stump of A4 + cystic artery – r‐RHA) and A4 stump, which was clamped with a vascular clip. d‐, donor's; r‐RHA, recipient's right hepatic artery

A 1‐year‐old boy with end‐stage Alagille syndrome was admitted to our hospital for evaluation as a candidate for LDLT. On admission, the patient's pediatric end‐stage liver disease score was 18, and his mother, aged 43 years, was the only living donor candidate. The mother's height and body weight were 156 cm and 51 kg, respectively. Multidetector row computed tomography (CT) (Aquilion Precision™; Toshiba) was used for preoperative dynamic CT evaluation. We performed a simulation of the donor hepatectomy, which was performed using SYNAPSE VINCENT three‐dimensional CT software (Fujifilm). The predicted left lateral segment graft volume was 196 mL, which was sufficiently large for the pediatric recipient because the graft–recipient weight ratio was 2.37%. Three‐dimensional CT angiography revealed that A2 originated separately from the left gastric artery, A3 + A4 originated from the left hepatic artery, and the cystic artery originated from A4 (Figure 1A). In the donor operation, we harvested the left lateral segment graft with A2 and A3 + A4 with the cystic artery stump (Figure 1B). Each orifice was approximately 1 mm in inner diameter, which was considered too small to obtain secure anastomosis in the recipient in situ. Therefore, we planned back‐table microsurgical hepatic artery angioplasty to combine the multiple hepatic artery orifices into one orifice. In this case, we considered that the arteries might kink or twist after hepatic artery reconstruction, if the cystic artery stump, which was turned over, was anastomosed with the A2 stump (Figure 1C). In detail, the graft had two orifices (one was the A2 orifice, and the other was the origin of A3, A4, and the cystic artery), but these orifices were not used as is, for anastomosis. We divided A4 at the proximal cystic artery branching (stump of the original artery for A3, A4, and the cystic artery; Figure 1C), and anastomosed the proximal stump orifice of A4 and the A2 orifice first, and then the origin artery for A3, A4, and the cystic artery, and the newly anastomosed A2 (proximal stump of A4, cystic artery) was used for anastomosis with the recipient's hepatic artery (Figure 1D).

We sacrificed A4 by dissecting the proximal conduit to the bifurcation of the cystic artery, and we ligated the distal stump of A4 because of the left lateral graft and the original artery of A3, A4, cystic artery stump was anastomosed with the A2 stump in an interrupted pattern under the surgical microscope during back‐table angioplasty, to avoid redundancy in the shape of A2 and A3 after angioplasty. We anastomosed the A3 + A4 conduit (original artery for A3, A4, and the cystic artery stump) to A2 to replace the hepatic artery because the caliber of the left gastric artery was not a size match to the A3 + A4 conduit, and the length of A2 was sufficient to anastomose to the A3 + A4. In the recipient, the single stump of the graft was anastomosed to the recipient's right hepatic artery, which was approximately 1 mm in diameter, using an interrupted pattern under the surgical microscope (Figure 1E). It took 19 minutes for ex vivo‐ and 32 minutes for in situ hepatic artery reconstruction. Total arterial reconstruction time was 51 minutes. The flow in the reconstructed artery was 110 mL/min.

### 2.2 Case presentation 2

**Figure 2:**
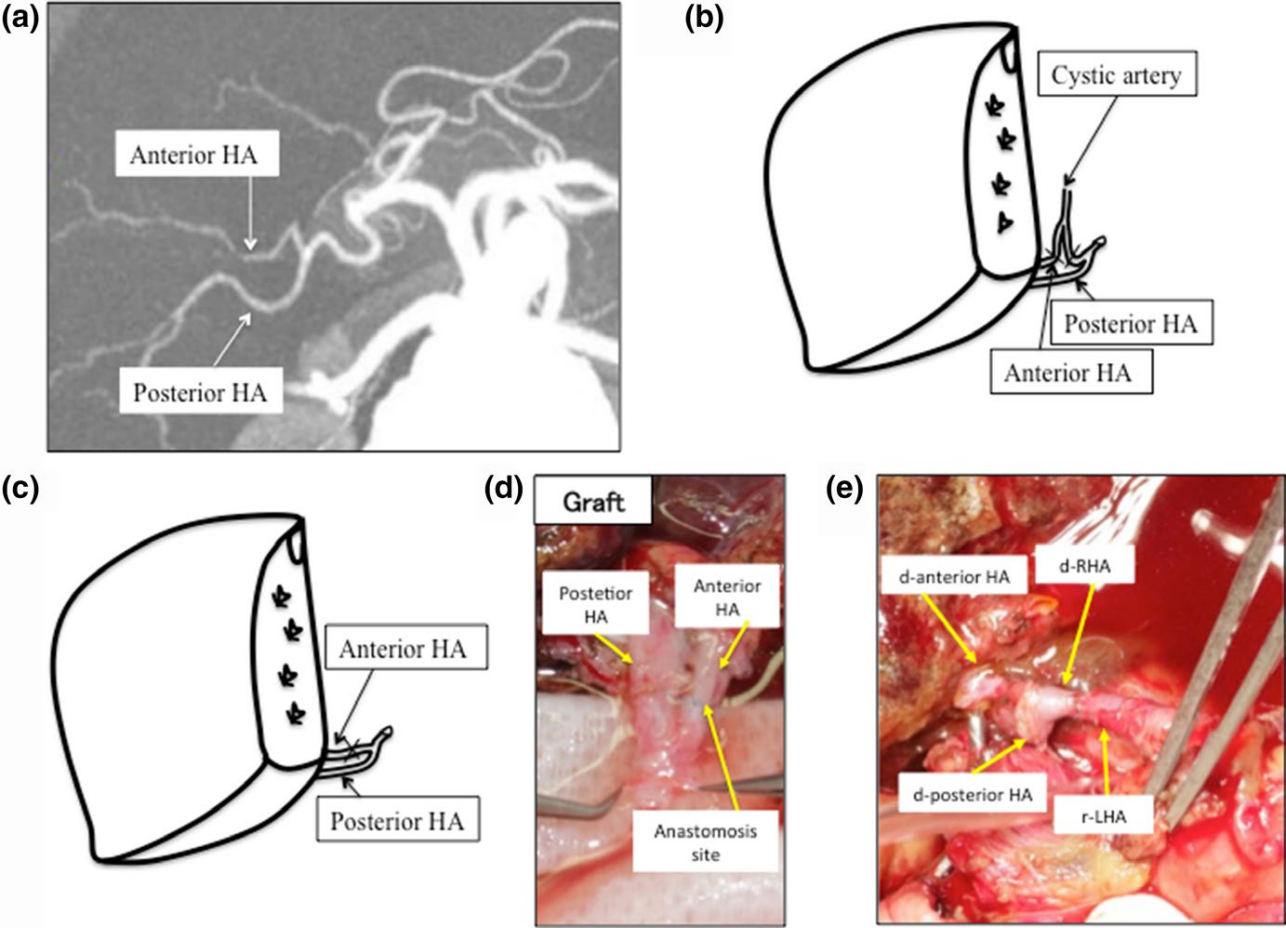
Imagings and schemas for Case 2. A, Three‐dimensional CT angiography of the hepatic artery (maximum intensity projection: MIP). Anterior HA, anterior hepatic artery; Posterior HA, posterior hepatic artery. B, Schema for the hepatic artery of the hepatic graft. Each line indicates the dividing point of the anterior hepatic artery. C, Schema for the graft after angioplasty of the anterior hepatic artery. The lines across the vessels indicate the back‐table angioplasty points under the surgical microscope. D, Photograph showing the back‐table angioplasty procedure for the hepatic artery. E, Intraoperative photograph of the reconstructed hepatic artery. d‐, donor’s; r‐LHA, recipient’s left hepatic artery

A 31‐year‐old man with acute on chronic liver failure secondary to alcohol was emergently admitted to our hospital for evaluation as a candidate for LDLT. On admission, his model for end‐stage liver disease score was 22, and his mother, aged 52 years, was the living donor candidate. Her height and body weight were 156 cm and 51 kg, respectively, and the recipient's height and body weight were 157 cm and 52 kg, respectively. Multidetector row CT was used for preoperative dynamic CT. Figure 2A shows a simulation of the donor hepatectomy, which was performed using the SYNAPSE VINCENT three‐dimensional CT software (Fujifilm). The predicted right‐ and left‐lobe volume was 635 and 379 mL, respectively. The right‐lobe graft was sufficiently large for the recipient, with a graft–recipient weight ratio of 1.22%, while the left‐lobe graft was small, with a ratio of 0.72%. Three‐dimensional CT angiography revealed that the anterior and posterior arteries originated separately from the proper hepatic artery (Figure 2A). It was impossible to procure the right‐lobe graft with single right hepatic artery because the anterior and posterior hepatic arteries encircled the common hepatic duct. Moreover, because the anterior artery travelled a deep roundabout route toward the infundibulum of the gall bladder and there was a severe adhesion around the anterior artery, we could not separate the anterior hepatic artery from the cystic artery (Figure 2B). Even if we could have separated the anterior artery from the cystic artery, we considered that the graft artery of the anterior artery was redundant and inappropriate for the reconstruction. Therefore, we planned to divide the anterior artery and to anastomose each anterior artery stump to avoid redundancy, using the surgical microscope, during back‐table angioplasty (Figure 2C, D). Figure 2E shows the microsurgical angioplasty of the anterior artery and the anastomosis of the recipient's left hepatic artery. It took 9 minutes for ex vivo‐ and 35 minutes for ex vivo hepatic artery reconstruction. Total arterial reconstruction time was 44 minutes. Flow in the reconstructed artery was 56 mL/min, and the flow shapes in the anterior and posterior artery were confirmed as good using Doppler ultrasonography.

## 3 SURGICAL PROCEDURES

This angioplasty procedure of the multiple graft hepatic arteries is performed under microsurgical scope. After the procurement of living donor graft, typically, donor organs are kept at a static temperature of 4°C in preservation solution. After portal vein perfusion, the microsurgeon reconstructed the hepatic artery with continuous zoom magnification of approximately × 10 using a surgical microscope (Carl Zeiss) in cold storage. The arterial anastomoses were performed using 9‐0 monofilament suture (Prolene; Ethicon, Inc.). We used a double‐needle suture and an interrupted pattern for the two hepatic artery stumps, with a biangular stay‐suture method. Each suture was placed from the inner side of the arterial wall to the outer side, which was safe and reliable. After placing the interrupted sutures in the anterior wall, we turned over the vessel in the cold storage on the back table, and placed interrupted sutures in the posterior wall. We placed a sprint tube (Atom multi‐use tube, 4 Fr; Atom Medical Corp.) into the reconstructed hepatic artery from the stump to prevent back wall suturing in ex vivo angioplasty. The most important points in hepatic arterial reconstruction in LDLT using this angioplasty are correct anatomical reconstruction of all hepatic arteries in the partial graft and anastomosing the hepatic arteries between the donor and recipient, while taking care of size mismatch, kinking, and redundant anastomosis of the reconstructed artery.

## 4 RESULTS

**Figure 3:**
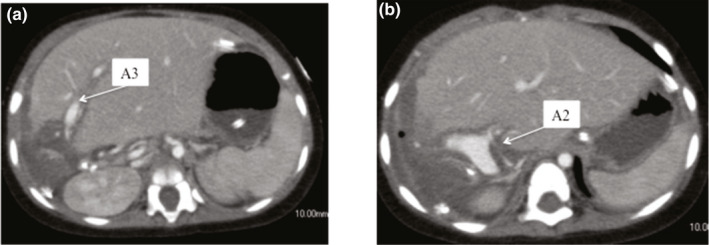
Postoperative CT images in Case 1. A, CT image of the reconstructed hepatic artery (A3) on postoperative day 7. B, CT image of the reconstructed hepatic artery (A2) on postoperative day 7

In case 1, the patency of A2 and A3 was confirmed by CT on postoperative day 7 (Figure 3A, B). The flow shape in the reconstructed artery was confirmed as good using Doppler ultrasonography until the postoperative day 14. The postoperative course of the donor and recipient was uneventful, and they were discharged on postoperative days 8 and 52, respectively. The case 1 recipient survived for 30 months, the reconstructed HA was patent on outpatient, and there were no symptoms of biliary complications.

**Figure 4:**
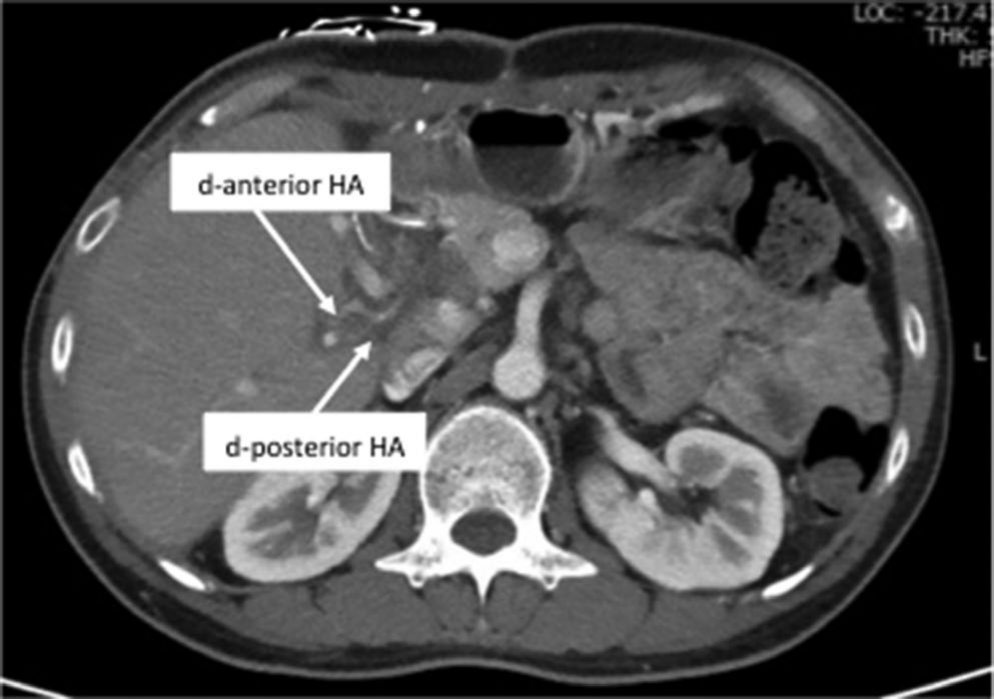
Postoperative CT images in Case 2. A, CT image of the reconstructed hepatic artery (anterior and posterior hepatic artery) on postoperative day 7

In case 2, the patency in the anterior and posterior hepatic artery was confirmed using CT on postoperative day 7 (Figure 4A). The postoperative course of the donor and recipient was uneventful, and they were discharged on postoperative days 10 and 18, respectively. The case 2 recipient survived for 6 months, the reconstructed HA was patent on outpatient, and there were no symptoms of biliary complications.

## 5 DISCUSSION

Hepatic artery reconstruction is one of the most complex procedures in LDLT because of the smaller arterial diameter, shorter stump, and increased risk of hepatic artery‐related complications, which lead to pivotal complications.

We currently perform hepatic artery reconstruction with interrupted sutures using a surgical microscope. Tanaka et al,^6^ in their initial experience, described favorable long‐term outcomes after left‐lobe LDLT when anastomosing the hepatic artery using an operating microscope. The authors concluded that the patency rates after microsurgical techniques were better, and the risk of hepatic artery thrombosis decreased significantly. The introduction of microsurgery dramatically decreased complication rates in hepatic artery reconstruction to between 0% and 6%.^7^

Although the microsurgical approach appears time‐consuming and complicated, it is the best approach in pediatric patients and patients with a small‐caliber hepatic artery of <2 mm.^8^, ^9^, ^10^, ^11^ Until now, we have performed 800 LDLTs, constituting 135 pediatric and 685 adult LDLTs, and hepatic artery‐related complications such as hepatic artery thrombosis occurred in 12 patients (1.5%). Moreover, more than 40% of left‐lobe grafts and 5% of right‐lobe grafts have multiple hepatic arteries, which creates a dilemma regarding which or both stumps should be reconstructed.^12^ Generally, back‐bleeding from a recessive arterial stump after reconstructing a dominant stump is the most important clue indicating anastomosis or ligation of a hepatic artery. However, Uchiyama et al reported that using grafts with double or triple arteries yielded favorable outcomes, with minimum artery‐related complications compared with grafts with a single artery.^12^, ^13^, ^14^ We recently reported that middle and left hepatic arterial reconstruction is safe in LDLT and may prevent biliary stricture caused by dual hepatic arterial reconstruction, when the graft has left and middle hepatic artery stumps.^5^ We recommend multi‐vessel anastomosis whenever feasible with left lateral segment grafts and right‐lobe grafts because identifying the dominant stump can be difficult. Dual or triple arterial stumps have similar sizes, making it difficult to appreciate the dominant stump.

Regarding left‐lobe grafts, we also recommend both middle and left hepatic arterial reconstruction to prevent biliary stricture. However, multi‐vessel hepatic arterial reconstruction requires more time to reconstruct multiple arteries and is often complicated because there is not always an adequately sized matching inflow artery in the recipient for multi‐vessel arterial grafting. For these reasons, we recommend back‐table microsurgical angioplasty to combine the two hepatic artery stumps into one orifice followed by sequential single hepatic artery reconstruction in the recipient. Lee et al also reported that back‐table angioplasty to combine the two hepatic artery stumps into one orifice was easy and safe in the right‐lobe graft during LDLT.^15^ We performed hepatic artery angioplasty in cold storage after portal vein reperfusion. In case 1 and case 2, it took 19 minutes and 9 minutes, respectively, for hepatic artery angioplasty. In both cases, we completed back‐table hepatic artery angioplasty before the recipient hepatectomy procedures. Therefore, cold ischemia time or anhepatic time was not prolonged, and the angioplasty procedure did not affect the graft quality regarding the cold ischemia time or anhepatic time. Moreover, it was thought that it took shorter time to reconstruct a single hepatic artery reconstruction after back‐table angioplasty than to reconstruct multiple hepatic arteries in situ. The most important merit of back‐table hepatic arterial angioplasty is achieving perfect stability of the flat and shallow surgical field on the back table under high magnification, without interference from respiratory and cardiac movement. The second important merit in back‐table hepatic arterial angioplasty is achieving safer and more reliable interrupted sutures compared with in situ multi‐vessel hepatic arterial reconstruction because we can freely change the suturing angle of the angioplasty vessels on the back table. Liang et al reported that back‐table microsurgical plasty should be used for grafts with arterial variation to minimize operative difficulties and to avoid arterial complications in LDLT.^16^

In our institute, sequential hepatic artery reconstruction after hepatic vein and portal vein reconstruction or donor graft harvest is performed by the same surgeon performing the angioplasty of the hepatic artery of the graft, while carefully considering size mismatch, kinking, and redundant anastomosis. The authors of a previous study found that the ideal hepatic arterial reconstruction was a short and non‐redundant anastomosis fashioned between the recipient and donor hepatic arteries. The authors demonstrated that using a long graft artery was an independent risk factor for early hepatic artery thrombosis.^17^

We use an interrupted anastomosis pattern. As a consideration, multi‐vessel hepatic arterial reconstruction may rescue graft arterial flow when one artery becomes thrombosed. However, we have no experience with rescue cases with multi‐vessel hepatic arterial reconstruction, judging the patency of the reconstructed hepatic artery according to the postoperative CT imaging.

The most important points in hepatic arterial reconstruction in LDLT are non‐redundant and non‐kinking anatomical reconstruction of all hepatic arteries in the partial graft, and anastomosing the hepatic arteries between the donor and recipient to prevent hepatic artery thrombosis.

In conclusion, back‐table microsurgical angioplasty is feasible for complex multi‐vessel arterial reconstruction in LDLT. We recommend performing secure multi‐vessel hepatic arterial reconstruction by adapting to the clinical scenario. By selecting a simple appropriate anastomosis, back‐table microsurgical angiography may provide good results in LDLT.

## 6 ACKNOWLEDGEMENT

We thank Jane Charbonneau, DVM, from Edanz Group (www.edanzediting.com/ac) for editing a draft of this manuscript.

## DISCLOSURE

Conflict of Interest: Authors declare no conflict of interests for this article.
